# A checklist of the amphibians and reptiles of Sinaloa, Mexico with a conservation status summary and comparisons with neighboring states

**DOI:** 10.3897/zookeys.931.50922

**Published:** 2020-04-30

**Authors:** Julio A. Lemos-Espinal, Geoffrey R. Smith

**Affiliations:** 1 Laboratorio de Ecología-UBIPRO, FES Iztacala UNAM, Avenida los Barrios 1, Los Reyes Iztacala, Tlalnepantla,edo. de Mexico, 54090, Mexico National Autonomous University of Mexico (UNAM) Tlalnepantla Mexico; 2 Department of Biology, Denison University, Granville, Ohio 43023, USA Denison University Granville United States of America

**Keywords:** checklist, crocodilians, frogs, herpetofauna, lizards, salamanders, snakes, turtles

## Abstract

Sinaloa possesses a rich biota with unique characteristics due to its proximity to the northern deserts, the tropical lowlands of the south, and the temperate environments of the western slopes of the Sierra Madre Occidental in Mexico. However, threats to its environment makes understanding the biological diversity of Sinaloa crucial. A checklist of the amphibians and reptiles has been generated, and the conservation status of Sinaloa’s herpetofauna summarized with the aim of understanding the potential conservation or management needs. Sinaloa has 159 species of amphibians and reptiles, including 39 species of amphibians and 120 species of reptiles. The herpetofauna of Sinaloa has relatively few species of conservation concern at a global and national scale (IUCN and SEMARNAT lists), but Environmental Vulnerability Scores suggest that there might be greater conservation concerns for the Sinaloa herpetofauna. Families of particular conservation concern include Craugastoridae, Eleutherodactylidae, Ambystomatidae, Crocodylidae, Dactyloidae, Eublepharidae, Helodermatidae, Iguanidae, Phrynosomatidae, Phyllodactylidae, Colubridae, Natricidae, Viperidae, Cheloniidae, and Dermochelyidae.

## Introduction

**Figure 1.**
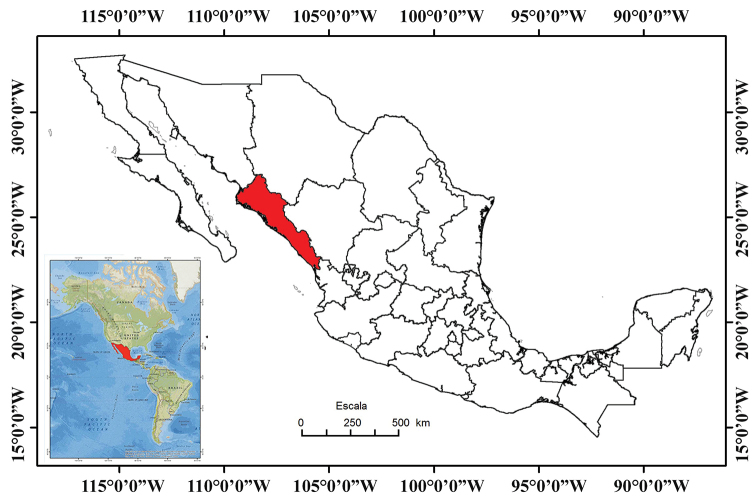
Map of Mexico with the state of Sinaloa shown in red (modified from INEGI 2018a).

The geographic position of Sinaloa (Fig. 1) results in a rich biota with unique characteristics which is composed of a mixture of species from the northern deserts, the tropical lowlands of the south, and the temperate environments of the western slopes of the Sierra Madre Occidental (see Bezy et al. 2017 for herpetofauna). Unfortunately, Sinaloa’s biological diversity is currently at risk. The growing human population of Sinaloa, which demands more and more resources, has created a large number of open landfills and increased air and water pollution from the use of unsustainable practices without any regard for and enforcement of environmental legislation (Beltrán 2017). Deforestation in Sinaloa has been particularly devastating as more than 50% of its surface area has been cleared to create cultivated areas, so that natural vegetation is now limited to isolated areas with limited access (INEGI 2017). Mangrove wetlands in Sinaloa are also being lost due to human activities (Manzano-Sarabia et al. 2018). In addition, there are numerous, more specific, threats to the herpetofauna of Sinaloa. These threats include the potential spread of emerging diseases of amphibians and reptiles (Mejia-Radillo et al. 2019; Saucedo et al. 2019), lowering or disruption of freshwater aquifers due to agricultural or residential use (Quinones et al. 1999; Torres-Sombra et al. 2013), heavy metal pollution from mining activities (Muñoz Sevilla et al. 2017), loss of natural land cover due to agricultural expansion (Ruíz-Luna and Berlanga-Robles 1999). This environmental degradation and increasing environmental threats make understanding the biological diversity of Sinaloa crucial. To that end, we contribute to the knowledge of the herpetofauna of Sinaloa by placing a checklist of the amphibians and reptiles in an easily accessible place. A previous checklist by Hardy and McDiarmid (1969) reported 131 species: 32 anurans, 33 lizards, 55 snakes, and ten turtles, and pointed out that the list might increase if access to the eastern mountains was improved. However, in recent years, there has been a paucity of studies on the herpetofauna of Sinaloa, in part due to the lack of security that prevails in the eastern part of the state where illegal crops are common, and to the lack of roads allowing access. We hope an updated checklist will provide a starting place for further research on the herpetofauna of Sinaloa. In addition, we summarize the conservation status of Sinaloa’s herpetofauna and compare the lists of amphibian and reptile species to those in neighboring states to identify unique aspects of the herpetofauna of Sinaloa, as well as shared species, with the aim of understanding the potential conservation or management needs at the state or regional level.

### Physiographic characteristics of the state

**Figure 2.**
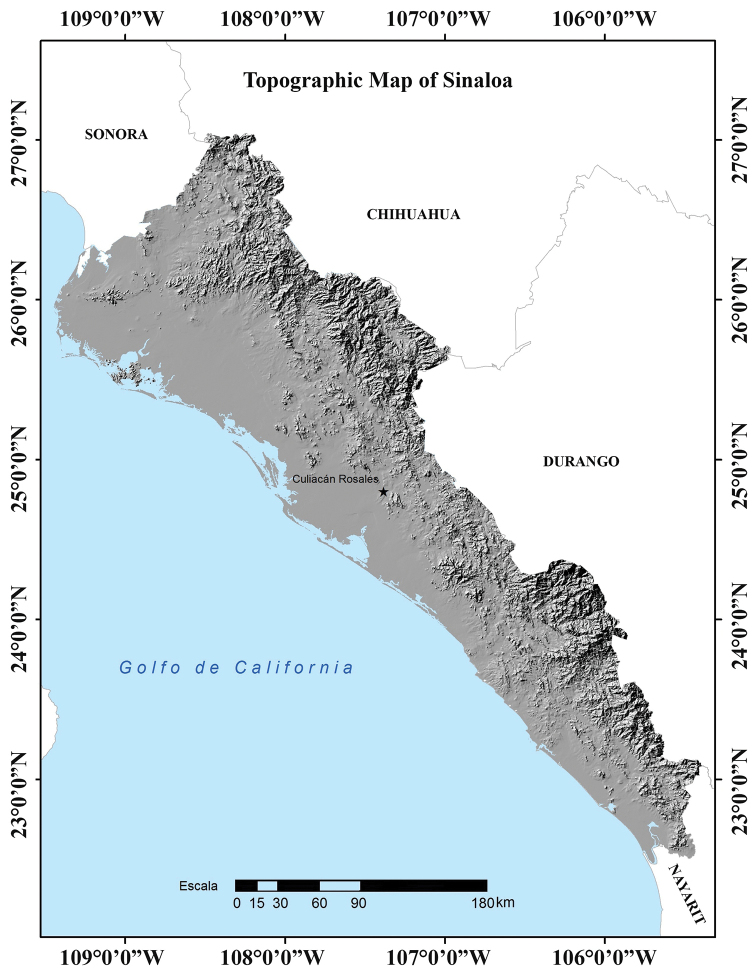
Topographical map of the state of Sinaloa, Mexico (INEGI 2009).

The relatively small state of Sinaloa (surface area of 58,328 km^2^) is located in northwestern Mexico, between 27°2'32" and 22°28'2"N and 105°23'32" and 109°26'52"W (Figs 1, 2; INEGI 2017). Sinaloa is bordered by Sonora to the north, Chihuahua and Durango to the east, Nayarit to the south, and the Gulf of California to the west.

The topography of Sinaloa can be divided into three large longitudinal strips (INEGI 2017). The first includes the mountain ranges of the Sierra Madre Occidental on the eastern side of the state. In Sinaloa, elevations rarely exceed 2,500 m, with the highest elevations near the border with Chihuahua (Cerro La Bandera: 2,280 m and Cerro Pelón: 2,500 m) and Durango (Cerro Alto: 2,800 m; Cerro Narizón: 2,560 m; and Cordón El Copo Alto: 2,360 m). The second strip is an extensive plain that lies between the foothills of the Sierra Madre Occidental and the Pacific Coast, which is the third strip. In northern Sinaloa the distance between the foothills of the Sierra Madre Occidental and the coast of the Pacific Ocean is greater than in southern Sinaloa, where they can be separated by less than 30 km. Throughout the state, the Pacific coastline is interrupted by large lagoons and mangroves, and although the coastline is straight and low, except for Mazatlán Bay, access to it is difficult due to the presence of these lagoons (Fig. 2; García-Martínez 2008; INEGI 2017).

**Figure 3.**
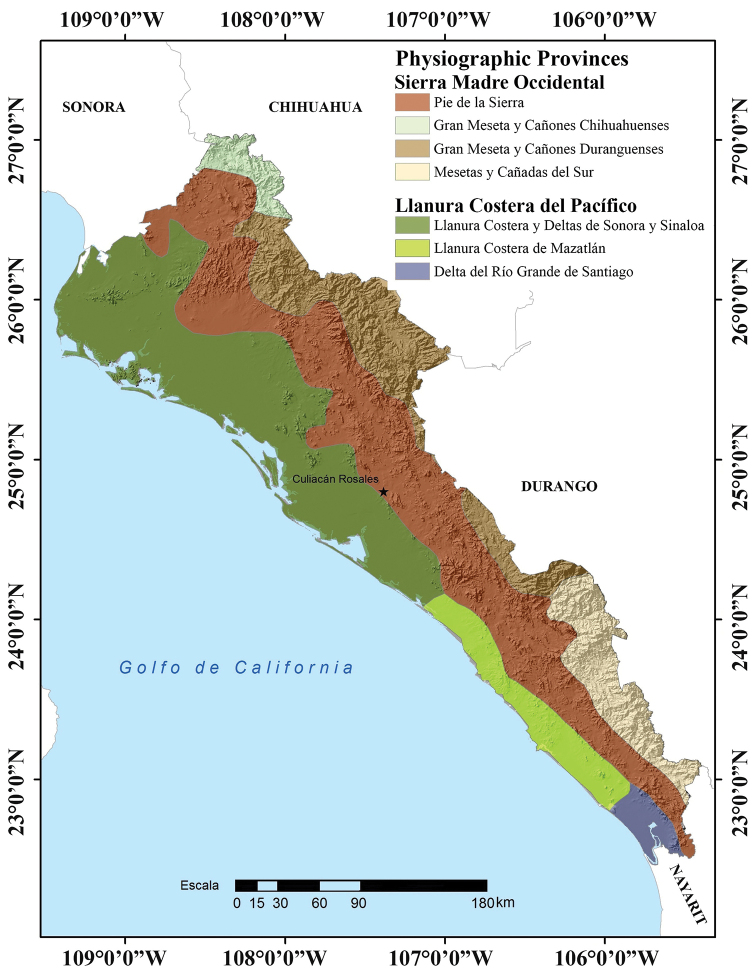
Physiographic provinces of the state of Sinaloa, Mexico (modified from Cervantes-Zamora et al. 1990).

Sinaloa includes two physiographic provinces: Sierra Madre Occidental and Llanura Costera del Pacífico. The Sierra Madre Occidental covers 59.5% of Sinaloa, covering a little more than the eastern half of the state (Fig. 3; INEGI 2017). The Llanura Costera del Pacífico covers 40.5% of the state, including almost all of the western half of the state (Fig. 3; INEGI 2017).

**Figure 4.**
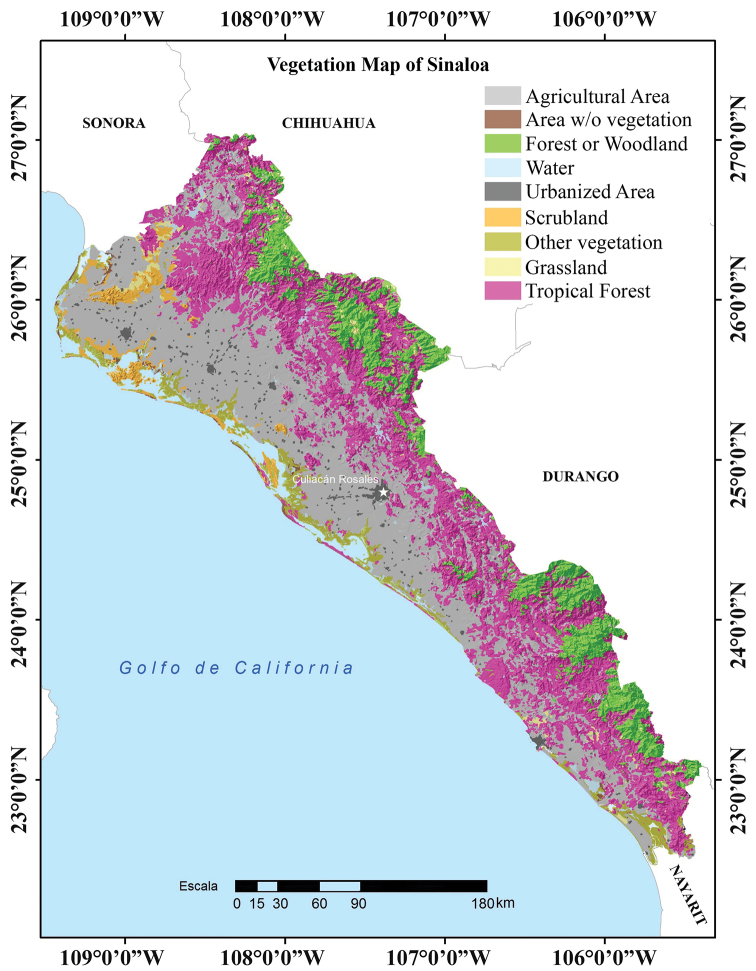
Vegetation map of the state of Sinaloa, Mexico (modified from Dirección General de Geografía – INEGI 2013).

The elongated shape of Sinaloa along with its topography characterized by a continuous mountain chain in the east running parallel to the coastline, produces a striped distribution of vegetation types in the state (Fig. 4). The flood plains of the main Sinaloa rivers and adjacent upland slopes have been cleared and cultivated for many centuries, such that the natural plant associations have been considerably altered, and the actual Sinaloa landscape has large areas with an unnaturally high percentage of commercially worthless trees and shrubs, and commercial crops such as corn, sorghum, tomatoes, mango, and sugarcane. The natural vegetation has been replaced by large areas of cultivation (Fig. 4), which also house numerous human populations ranging from small ejidos to large cities (Brand 1936; INEGI 2017). The dominant natural vegetation is tropical deciduous forest found along the western slopes of the Sierra Madre Occidental of Sinaloa. Tropical deciduous forest in southern Sinaloa is separated from the upland oak woodland and pine-oak forest by semi-deciduous tropical forest, a much more tropical vegetation type (Ruíz-Guerrero et al. 2015). The density of this forest is higher in the southern third of Sinaloa and is more open in the northeastern part of the state. In addition, southern Sinaloa has the huge Marisas Nacionales wetlands that abut semi-deciduous forest (T. Van Devender, pers. comm.). Tree species in the southern third are also taller than those in the northern part of the state. This vegetation type is found from the southern third of the state along the foothills of the Sierra Madre Occidental to the northeastern corner of the state. On the highest mountains of the Sierra Madre Occidental, the vegetation often changes to oak and pine-oak forest along the borders with Durango and Chihuahua (INEGI 2017). The coastal plain of the northern half of Sinaloa shows great uniformity in vegetation, gradually changing as one moves to the south (INEGI 2017). The belt immediately along the coast is more arid than the interior and its vegetation is poorer and more open. The flood plains are largely devoted to vast cultivated fields of sugar, garbanzo, tomatoes, and corn. The natural vegetation is more luxuriant than that of the uplands and includes many tropical plants. The vegetation of the hills, which are scattered over the plain, is commonly very similar to that of the plain. In the lowlands of Sinaloa, the coastal plain type of “thorn forest”, a mixture of tropical deciduous forest and thornscrub, predominates in area over the vegetation characterizing the flood plains, the coast, and the hills (Shreve 1937). The vegetation along the coast of Sinaloa north of Mazatlán is a vegetation type that could be considered short tropical deciduous forest (T. Van Devender, pers. comm.). In the northwestern corner of the state near Sonora, the vegetation type is subtropical Mimosaceae-cacti characterized by spiny shrubs and cacti dominated by Mimosaceae and columnar cacti. This vegetation is not distributed uniformly, rather it is arranged in clumps, but with a nearly continuous cover in wetter spots (Brand 1936). In Sonora, thorn scrub is a transitional vegetation type between tropical deciduous forest and the Sonoran Desert to the north and the woodlands and forests of the Sierra Madre Occidental to the east (Martin et al. 1998; Van Devender et al. 2013).

**Figure 5.**
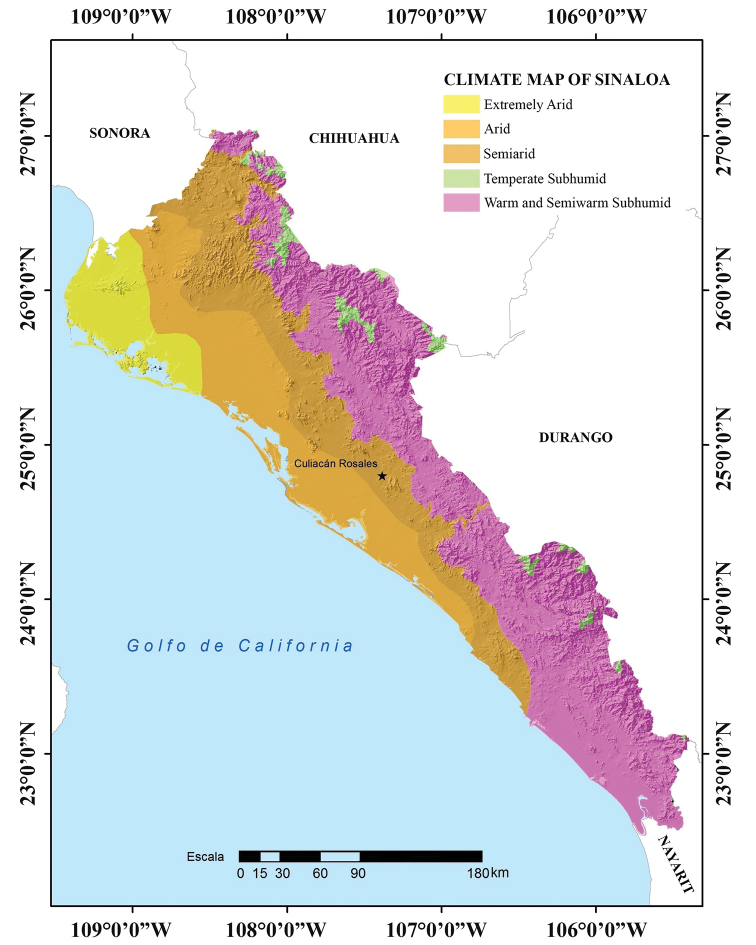
Climate map of the state of Sinaloa, Mexico (modified from García – Comisión Nacional para el Conocimiento y Uso de la Biodiversidad 1998).

In Sinaloa there is a trend for precipitation to decrease from southern to northern Sinaloa. The dominant climate in Sinaloa is warm semi-warm sub-humid which covers 48.4% of the state and is present from the southern tip of the state to the Port of Mazatlán, and from there in a narrow strip along the foothills of the western slopes of the Sierra Madre Occidental of Sinaloa, along the borders with Durango and Chihuahua. This area is characterized by a mean annual temperature over 18 °C. Precipitation of the driest month is < 40 mm. Small scattered locations in the highest mountains of the extreme southeastern and northeastern parts of Sinaloa are characterized by a temperate sub-humid climate, present in only 2.3% of the state. The climate of these elevated peaks is characterized by an average annual temperature between 12 °C and 18 °C. Rainfall in the driest month is < 40 mm; the maximum rainfall occurs in summer. A narrow strip that runs parallel to the foothills of the Sierra Madre Occidental, covering 21.3% of the state surface area, from just north of the Port of Mazatlán to the border with Sonora in northern Sinaloa is characterized by a semiarid climate with a mean annual temperature > 22 °C. Parallel to this strip and next to the coastline the climate is arid, with an average annual temperature > 22 °C. The extreme northwestern corner of the state, from the border with Sonora to just south of the Port of Topolobampo, which covers 9.8% of the state surface territory, is characterized by an extreme arid climate with an average annual temperature above 22 °C, and eight continuous months of dry to very dry conditions. Heavy rains occur in the July-September, which represent more than 75% of the annual total (Fig. 5; Köppen modified by García 1998; INEGI 2017).

## Materials and methods

We compiled this list of amphibians and reptiles of Sinaloa based on our field work, a thorough examination of the available literature on amphibians and reptiles in Sinaloa, and records of amphibians and reptiles from Sinaloa in VertNet.org. We only included species if we were able to confirm records, either by direct observation or through documented museum records or vouchers. We follow Frost (2019) and AmphibiaWeb (2019) (http://amphibiaweb.org) for amphibian names and Uetz and Hošek (2019) for reptile names. We generated species accumulation curves the total herpetofauna, amphibians, and reptiles using the year of the first recorded observation for each species. Such curves reasonably estimate potential species richness of amphibians and reptiles (Raxworthy et al. 2012). We determined the conservation status of each species from the IUCN Red List 2019-2 (IUCN 2019), SEMARNAT (2010), and Environmental Vulnerability Scores (Wilson et al. 2013a, b; Johnson et al. 2015). We determined the number of species found in Sinaloa that were shared with the four neighboring states using recent herpetofaunal check lists for Sonora (Lemos-Espinal et al., 2019a), Chihuahua (Lemos-Espinal et al. 2017), Durango (Lemos-Espinal et al. 2019b), and Nayarit (Woolrich-Piña et al. 2016). We also included the Baja California Peninsula in this comparison, using Grismer (2002) and Hollingsworth et al. (2015) as species lists, since it used to be contiguous with the states of Sinaloa and Sonora. To avoid overestimation of the shared species in this region we excluded species occurring only on Islands Tiburón and San Esteban, since those are included in the Sonora Checklist but not the Peninsula proper.

## Results and discussion

Sinaloa is home to 159 species of amphibians and reptiles representing 35 families (including two introduced: Gekkonidae and Typhlopidae) and 89 genera (including three introduced: *Gehyra*, *Hemidactylus*, and *Indotyphlops*) (Table 1). There are 39 species of amphibians (38 anurans [one introduced], and one salamander) and 120 reptiles (one crocodilian, 42 lizards [two introduced], 64 snakes [one introduced], and 13 turtles). The four introduced species are: the American Bullfrog (*Ranacatesbeiana*), the Stump-toed Gecko (*Gehyramutilata*), the Common House Gecko (*Hemidactylusfrenatus*), and the Brahminy Blindsnake (*Indotyphlopsbraminus*). *Anolisutowanae* is the only species endemic to Sinaloa and is only known from the type specimen. There are six marine species that occur along the coast of Sinaloa (*Hydrophisplaturus*, *Carettacaretta*, *Cheloniamydas*, *Eretmochelysimbricata*, *Lepidochelysolivacea*, and *Dermochelyscoriacea*).

**Table 1.** Amphibians and reptiles of Sinaloa with distributional and conservation status. Vegetation Type: 1 = Tropical Deciduous Forest; 2 = Thorn Forest; 3 = Subtropical Mimosaceae Cacti; 4 = Oak Forest; 5 = Pine-Oak Forest; 6 = Marine; IUCN Status: DD = Data Deficient; LC = Least Concern, VU = Vulnerable, NT = Near Threatened; EN = Endangered; CE = Critically Endangered; NE = not Evaluated according to the IUCN Red List (The IUCN Red List of Threatened Species, Version 2019-2 (www.iucnredlist.org; accessed 26 October 2019); Environmental Vulnerability Score: EVS – the higher the score the greater the vulnerability: low (L) vulnerability species (EVS of 3–9); medium (M) vulnerability species (EVS of 10–13); and high (H) vulnerability species (EVS of 14–20) from Wilson et al. (2013a, b) and Johnson et al. (2015); conservation status in Mexico according to SEMARNAT (2010): P = in danger of extinction, A = threatened, Pr = subject to special protection, NL – not listed; Global Distribution: 0 = Endemic to Sinaloa; 1 = Endemic to Mexico; 2 = Shared between the US and Mexico; 3 = widely distributed from Mexico to Central or South America; 4 = widely distributed from the US to Central or South America; 5 = circumglobal distribution; 6 = Pacific and Indian Oceans; IN = Introduced to Sinaloa. Date in which the first record appeared; and Source of the first record.

	Vegetation type	IUCN Status	EVS	SEMARNAT	Global distribution	Date of first record	Source
**Class Amphibia**							
**Order Anura**							
**Bufonidae**							
*Anaxyruscognatus* (Say, 1823)	3	LC	L (8)	NL	2	1974	UAZ Herpetology UAZ 38720
*Anaxyruskelloggi* (Taylor, 1936)	1, 2, 3, 4	LC	H (14)	NL	1	1936	Taylor 1936
*Anaxyrusmexicanus* (Brocchi, 1879)	4, 5	NT	M (13)	NL	1	2009	Enderson et al. 2009
*Anaxyruspunctatus* (Baird & Girard, 1852)	1, 2, 3, 4	LC	L (5)	NL	2	1934	FMNH Amphibians and Reptiles 102426
*Inciliusalvarius* (Girard, 1859)	1, 2, 3	LC	M (11)	NL	2	1953	MVZ:Herp:58724
*Inciliusmarmoreus* (Wiegmann, 1833)	1, 2, 4	LC	M (11)	NL	1	1925	CAS HERP 64980
*Inciliusmazatlanensis* (Taylor, 1940)	1, 2, 4	LC	M (12)	NL	1	1940	Taylor 1940
*Inciliusoccidentalis* (Camerano, 1879)	4, 5	LC	M (11)	NL	1	1946	MVZ:Herp:44692
*Rhinellahorribilis* (Wiegmann, 1833)	1, 2, 3, 4	NE	NE	NL	4	1905	UAZ Herpetology UAZ 55928
**Craugastoridae**							
*Craugastoraugusti* (Dugès, 1879)	1, 4	LC	L (8)	NL	2	1955	KU KUH 41556
*Craugastorhobartsmithi* (Taylor, 1937)	1, 4	EN	H (15)	NL	1	1963	KU KUH 75259
*Craugastoroccidentalis* (Taylor, 1941)	1, 4	DD	M (13)	NL	1	1897	USNM Amphibians & Reptiles 47433
*Craugastorpygmaeus* (Taylor, 1937)	1, 4	VU	L (9)	NL	1	1963	CAS HERP 175697
*Craugastorvocalis* (Taylor, 1940)	1, 4	LC	M (13)	NL	1	1955	KU KUH 41530
**Eleutherodactylidae**							
*Eleutherodactylusinterorbitalis* (Langebartel & Shannon, 1956)	1, 4	DD	H (15)	Pr	1	1955	USNM Amphibians & Reptiles 139727
*Eleutherodactylusnitidus* (Peters, 1870)	1, 4	LC	M (12)	NL	1	1962	LACM Herps 90544
*Eleutherodactylussaxatilis* (Webb, 1962)	4	EN	H (17)	NL	1	1961	KU KUH 63326
*Eleutherodactylusteretistes* (Duellman, 1958)	1, 4	DD	H (16)	Pr	1	1963	KU KUH 75264
**Hylidae**							
*Dryophytesarenicolor* (Cope, 1886)	1, 4, 5	LC	L (7)	NL	2	1920	USNM Amphibians & Reptiles 84411
*Dryophyteseximius* (Baird, 1854)	5	LC	M (10)	NL	1	2019	https://www.inaturalist.org/taxa/65551-Hyla-eximia
*Exerodontasmaragdina* (Taylor, 1940)	1, 4	LC	M (12)	Pr	1	1957	KU KUH 68719
*Sarcohylabistincta* (Cope, 1877)	1, 4	LC	L (9)	Pr	1	1955	KU KUH 44567
*Smiliscabaudinii* (Duméril & Bibron, 1841)	1, 2, 3, 4	LC	L (3)	NL	4	1955	Smith and Van Gelder 1955
*Smiliscafodiens* (Boulenger, 1882)	1, 2, 3, 4	LC	L (8)	NL	2	1882	Boulenger 1882
*Tlalocohylasmithii* (Boulenger, 1902)	1, 2, 4	LC	M (11)	NL	1	1953	UMMZ Herps 110915
*Trachycephalusvermiculatus* (Cope, 1877)	2	NE	L (4)	NL	3	1962	LACM Herps 6316
*Tripionspatulatus* Günther, 1882	1, 2	LC	M (13)	NL	1	1882	Günther 1882
**Leptodactylidae**							
*Leptodactylusmelanonotus* (Hallowell, 1861)	1, 2, 3, 4	LC	L (6)	NL	3	1894	CAS HERP 3161
**Microhylidae**							
*Gastrophrynemazatlanensis* (Taylor, 1943)	1, 2, 4	NE	L (8)	NL	2	1943	Taylor 1943
*Hypopachusustus* (Cope, 1866)	1, 2	LC	L (7)	Pr	3	1918	USNM Amphibians & Reptiles 73267
*Hypopachusvariolosus* (Cope, 1866)	1, 2	LC	L (4)	NL	4	1883	Boulenger 1883
**Phyllomedusidae**							
*Agalychnisdacnicolor* (Cope, 1864)	1, 2, 4	LC	M (13)	NL	1	1960	UF Herp 12855
**Ranidae**							
*Ranacatesbeiana* Shaw, 1802	**NA**	**NA**	**NA**	**NA**	**NA**	**IN**	
*Ranaforreri* Boulenger, 1883	1, 2, 4	LC	L (3)	Pr	3	1883	Boulenger 1883
*Ranamagnaocularis* Frost & Bagnara, 1976	1, 2, 3, 4	LC	M (12)	NL	1	1818	MVZ:Herp:175932
*Ranapustulosa* Boulenger, 1883	1, 2, 4	LC	L (3)	Pr	1	1953	MVZ:Herp:58962
*Ranatarahumarae* Boulenger, 1917	4, 5	VU	L (8)	NL	2	1985	UAZ Herpetology UAZ 46087
**Scaphiopodidae**							
*Scaphiopuscouchi* Baird, 1854	1, 2, 3, 4	LC	L (3)	NL	2	1970	UTEP:Herp:5902
**Order Caudata**							
**Ambystomatidae**							
*Ambystomarosaceum* Taylor, 1941	4, 5	LC	H (14)	Pr	1	1954	CAS SUA 18388
**Class Reptilia**							
**Order Crocodylia**							
**Crocodylidae**							
*Crocodylusacutus* Cuvier, 1807	1, 2	VU	H (14)	Pr	4	1912	LACM Herps 138123
**Order Squamata**							
**Suborder Lacertilia**							
**Anguidae**							
*Barisiaciliaris* (Smith, 1942)	4, 5	NE	H (15)	NL	1	1904	AMNH Herpetology R-585
*Elgariakingii* Gray, 1838	1, 4, 5	LC	M (10)	Pr	2	1963	KU KUH 78903
*Gerrhonotusliocephalus* Wiegmann, 1828	4	LC	L (6)	Pr	2	1961	UMMZ Herps 123044
**Dactyloidae**							
*Anolisnebulosus* (Wiegmann, 1834)	1, 2, 3, 4	LC	M (13)	NL	1	1834	Wiegmann 1834
*Anolisutowanae* Barbour, 1932	1	DD	H (17)	Pr	0	1932	Barbour 1932
**Eublepharidae**							
*Coleonyxfasciatus* (Boulenger, 1885)	1, 2	LC	H (17)	NL	1	1963	CAS HERP 115551
*Coleonyxvariegatus* (Baird, 1858)	3	LC	M (11)	Pr	2	1963	LACM Herps 93673
**Gekkonidae (Introduced)**							
*Geyhramutilata* (Wiegmann, 1834)	**NA**	**NA**	**NA**	**NA**	**NA**		
*Hemidactylusfrenatus* Schlegel, 1836	**NA**	**NA**	**NA**	**NA**	**NA**		
**Helodermatidae**							
*Helodermahorridum* Wiegmann, 1829	1, 2	LC	M (11)	A	3	1700	MCZ Herp R-7012
*Helodermasuspectum* Cope, 1869	3	NT	H (15)	A	2	1966	TNHC Herpetology 107291
**Iguanidae**							
*Ctenosauramacrolopha* Smith, 1972	1, 2	NE	H (19)	NL	1	1904	USNM Amphibians & Reptiles 33571
*Ctenosaurapectinata* (Wiegmann, 1834)	1, 2	NE	H (15)	NL	1	1886	Cope 1886
*Dipsosaurusdorsalis* (Baird & Girard, 1852)	3	LC	M (11)	NL	2	1933	LACM Herps 8646
*Iguanaiguana* (Linnaeus, 1758)	1, 2	LC	M (12)	Pr	3	1894	CAS SUR 2868
**Phrynosomatidae**							
*Callisaurusdraconoides* Blainville, 1835	1, 2, 3	LC	M (12)	A	2	1894	CAS HERP 3390
*Holbrookiaelegans* Bocourt, 1874	1, 2, 4	LC	M (13)	NL	2	1874	Bocourt 1874
*Phrynosomasolare* Gray, 1845	1, 2, 3, 4	LC	H (14)	NL	2	1898	USNM Amphibians & Reptiles 47541
*Sceloporusalbiventris* Smith, 1939	1, 2, 4	NE	H (16)	NL	1	1897	USNM Amphibians & Reptiles 47678
*Sceloporusbulleri* Boulenger, 1894	1, 4	LC	H (15)	NL	1	1946	MVZ:Herp:44695
*Sceloporusclarkii* Baird & Girard, 1852	1, 2, 3, 4	LC	M (10)	NL	2	1893	Stejneger 1893
*Sceloporusjarrovii* Cope, 1875	1, 4, 5	LC	M (11)	NL	2	1956	UAZ Herpetology UAZ 02688
*Sceloporusmagister* Hallowell, 1854	1, 2, 3	LC	L (9)	NL	2	1961	CM Herps 38193
*Sceloporusnelsoni* Cochran, 1923	1, 2	LC	M (13)	NL	1	1923	Cochran 1923
*Sceloporuspoinsettii* Baird & Girard, 1852	4, 5	LC	M (12)	NL	2	1954	LACM Herps 97377
*Sceloporusshannonorum* Langebartel, 1959	4	NE	H (15)	NL	1	1959	UCM:Herp:12951
*Sceloporusspinosus* Weigmann, 1828	1, 2, 4, 5	LC	M (12)	NL	1	1959	UCM:Herp:12949
*Sceloporusutiformis* Cope, 1864	1, 2	LC	H (15)	NL	1	1897	USNM Amphibians & Reptiles 47687
*Sceloporusvirgatus* Smith, 1938	4	LC	H (15)	NL	2	1969	CAS HERP 155905
*Urosaurusbicarinatus* (Duméril, 1856)	1, 2	LC	M (12)	NL	1	1934	FMNH Amphibians and Reptiles 106516
*Urosaurusornatus* (Baird & Girard, 1852)	1, 2	LC	M (10)	NL	2	1899	USNM Amphibians & Reptiles 46628
**Phyllodactylidae**							
*Phyllodactylushomolepidurus* Smith, 1935	1, 2	LC	H (15)	Pr	1	1964	LACM Herps 93782
*Phyllodactyluslanei* Smith, 1935	1, 2	LC	H (15)	NL	1	1936	Taylor 1936
*Phyllodactylustuberculosus* Wiegmann, 1835	1, 2	LC	L (8)	NL	3	1897	Van Denburgh 1897
**Scincidae**							
*Plestiodoncallicephalus* (Bocourt, 1879)	1, 2, 4	LC	M (12)	NL	2	1962	KU KUH 73745
*Plestiodoncolimensis* (Taylor 1935)	1, 2	DD	H (14)	Pr	1	1955	KU KUH 44733
*Plestiodonparviauriculatus* (Taylor, 1933)	1, 2, 4	DD	H (15)	Pr	1	1967	CAS HERP 155915
*Plestiodonparvulus* (Taylor, 1933)	2, 4	DD	H (15)	NL	1	1964	KU KUH 91415
**Teiidae**							
*Aspidosceliscommunis* (Cope, 1978)	1, 2	LC	H (14)	Pr	1	1897	Van Denburgh 1897
*Aspidosceliscostatus* (Cope, 1878)	1, 2, 3	LC	M (11)	Pr	1	1953	MVZ: Herp:59184
*Aspidoscelisstictogrammus* (Burger, 1950)	1, 2, 3	LC	H (14)	NL	2	1974	CAS HERP 222149
*Aspidoscelistigris* (Baird & Girard, 1852)	1, 2, 3	LC	L (8)	NL	2	1955	KU KUH 44724
**Order Squamata**							
**Suborder Serpentes**							
**Boidae**							
*Boasigma* Smith, 1943	1, 2, 3	NE	H (15)	NL	1	1898	USNM Amphibians & Reptiles 46503
**Colubridae**							
*Arizona elegans* Kennicott, 1859	1, 2	LC	L (5)	NL	1	1962	CAS HERP 93858
*Chilomeniscusstramineus* Cope, 1860	1, 2	LC	L (8)	Pr	2	1975	LACM Herps 121310
*Conopsisnasus* Günther, 1858	4, 5	LC	M (11)	NL	1	1963	CAS SUR 23795
*Drymarchonmelanurus* (Duméril, Bribon & Duméril, 1854)	1, 2, 3, 4	LC	L (6)	NL	4	1897	USNM Amphibians & Reptiles 46430
*Drymobiusmargaritiferus* (Schlegel, 1837)	1, 2	LC	L (6)	NL	4	1957	MSUM HE HE. 180
*Geagrasredimitus* Cope, 1875	1, 2	DD	H (14)	Pr	1	1936	Taylor 1936
*Gyalopionquadrangulare* (Günther, 1893)	1, 2, 3	LC	M (11)	Pr	2	1893	Günther 1893
*Lampropeltisgreeri* Webb, 1961	1, 4	NE	NE	NL	1	2009	Enderson et al. 2009
*Lampropeltisnigrita* Zweifel & Norris, 1955	1, 2, 3	NE	NE	NL	2	1961	LACM Herps 75333
*Lampropeltispolyzona* Cope, 1860	1, 2, 3	LC	L (7)	NL	1	1953	MVZ: Herp:59295
*Lampropeltiswebbi* Bryson, Dixon & Lazcano, 2005	4	DD	H (16)	NL	1	2005	Bryson et al. 2005
*Leptophisdiplotropis* (Günther, 1872)	1, 2, 3, 4	LC	H (14)	A	1	1897	Van Denburgh 1897
*Masticophisbilineatus* Jan, 1863	1, 2, 3	LC	M (11)	NL	2	|894	CAS HERP 3391
*Masticophisflagellum* Shaw, 1802	1, 2, 3	LC	L (8)	A	2	1904	USNM Amphibians & Reptiles 33570
*Masticophismentovarius* (Duméril, Bribon & Duméril, 1854	1, 2, 3, 4	LC	L (6)	A	3	1959	UAZ Herpetology UAZ 16305
*Mastigodryascliftoni* (Hardy, 1964)	1, 4	NE	H (14)	NL	1	1962	KU KUH 73490
*Mastigodryasmelanolomus* (Cope 1868)	1, 2	LC	L (6)	NL	3	1963	KU KUH 80746
*Oxybelisaeneus* (Wagler, 1824)	1, 2	NE	L (5)	NL	4	1925	CAS HERP 64981
*Phyllorhynchusbrowni* Stejneger, 1890	1, 2, 3	LC	M (13)	Pr	2	1954	KU KUH 37597
*Phyllorhynchusdecurtatus* (Cope, 1868)	1, 2, 3	LC	M (11)	NL	2	1962	KU KUH 73609
*Pituophiscatenifer* (Blainville, 1835)	1, 2, 3, 4	LC	L (9)	NL	2	1953	MVZ: Herp:59289
*Pituophisdeppei* (Dumeril, 1853)	4	LC	H (14)	A	1	1975	LACM Herps 136856
*Pseudoficimiafrontalis* (Cope, 1864)	1, 2	LC	M (13)	NL	1	1958	LACM Herps 103652
*Rhinocheiluslecontei* Baird & Girard, 1853	1, 2	LC	L (8)	NL	2	1956	UMMZ Herps 114488
*Salvadorabairdii* Jan & Sordelli, 1860	1, 2, 4	LC	H (15)	Pr	1	1961	MSUM HE HE. 11367
*Salvadoradeserticola* Schmidt, 1940	1, 2, 3	NE	H (14)	NL	2	1910	Smith 1941
*Salvadorahexalepis* (Cope, 1867)	1, 2, 3	LC	M (10)	NL	2	1962	KU KUH 73627
*Senticolistriaspis* (Cope, 1866)	1, 2	LC	L (6)	NL	4	1960	LACM Herps 103798
*Sonoraaemula* (Cope, 1879)	1, 2, 4	NT	H (16)	Pr	1	1956	UAZ Herpetology UAZ 16533
*Sonoramutabilis* Stickel, 1943	1, 2	LC	H (14)	NL	1	??	UTA UTA-R 7227
*Sympholislippiens* Cope, 1862	1, 2	NE	H (14)	NL	1	1960	LACM Herps 103696
*Tantillabocourti* (Günther, 1895)	1, 4	LC	L (9)	NL	1	1968	CAS HERP 155923
*Tantillacalamarina* Cope, 1866	2	LC	M (12)	Pr	1	1875	Cope 1875
*Tantillawilcoxi* Stejneger, 1902	4, 5	LC	M (10)	NL	2	1968	CAS HERP 155925
*Tantillayaquia* Smith, 1942	1, 2	LC	M (10)	NL	2	1925	CAS HERP 64976
*Trimorphodonpaucimaculatus* Taylor, 1936	1, 2, 3, 4	NE	H (15)	NL	1	1936	Taylor 1936
*Trimorphodontau* Cope, 1870	1, 2	LC	M (13)	NL	1	1953	FMNH Amphibians and Reptiles 71531
**Dipsadidae**							
*Coniophaneslateritius* Cope, 1862	1, 2	DD	M (13)	NL	1	1963	KU KUH 83401
*Diadophispunctatus* (Linnaeus, 1766)	4	LC	L (4)	NL	2	1964	UTEP:Herp:4026
*Geophisdugesii* Bocourt, 1883	4	LC	M (13)	NL	1	1972	CM Herps 69071
*Hypsiglenachlorophaea* Cope, 1860	1, 2, 3	NE	L (8)	NL	2	1956	TCWC Herpetology 12603
*Hypsiglenatorquata* (Günther, 1860)	1, 2	LC	L (8)	Pr	1	1894	CAS HERP 3394
*Imantodesgemmistratus* (Cope, 1861)	1, 2, 3	LC	L (6)	Pr	3	1956	UMMZ Herps 114466
*Leptodeiramaculata* (Hallowell, 1861)	1, 2	LC	L (7)	Pr	1	1918	USNM Amphibians & Reptiles 62201
*Leptodeirapunctata* (Peters, 1866)	1, 2, 3	LC	H (17)	NL	1	1897	Van Denburgh 1897
*Leptodeirasplendida* Günther, 1895	1, 2	LC	H (14)	NL	1	1897	USNM Amphibians & Reptiles 46459
*Rhadinaeahesperia* Bailey, 1940	1, 4	LC	M (10)	Pr	1	1897	USNM Amphibians & Reptiles 46456
*Tropidodipsasannulifera* (Boulenger, 1894)	1, 2	LC	M (13)	Pr	1	1960	LACM Herps 7115
*Tropidodipsasphilippi* (Jan, 1863)	1, 2	LC	H (14)	Pr	1	1962	KU KUH 73640
**Elapidae**							
*Hydrophisplaturus* (Linnaeus, 1766)	6	LC	NE	NL	6	1951	SDNHM Herps 41205
*Micruroideseuryxanthus* (Kennicott, 1860)	1, 2	LC	H (15)	A	2	1956	UMMZ Herps 114637
*Micrurusdistans* (Kennicott, 1860)	1, 2, 3	LC	H (14)	Pr	1	1962	LACM Herps 7187
**Leptotyphlopidae**							
*Renadugesii* (Bocourt, 1881)	1, 2	NE	NE	NL	1	1894	CAS SUR 1776
**Natricidae**							
*Storeriastorerioides* (Cope, 1865)	4, 5	LC	M (11)	NL	1	1961	UMMZ Herps 123036
*Thamnophiscyrtopsis* (Kennicott, 1860)	1, 2, 3, 4	LC	L (7)	A	4	1897	USNM Amphibians & Reptiles 46457
*Thamnophisvalidus* (Kennicott, 1860)	1, 2, 3	NE	M (12)	NL	1	1879	Fischer 1879
**Typhlopidae**							
*Indotyphlopsbraminus* (Daudin, 1803)	**NA**	**NA**	**NA**	**NA**	**NA**		
**Viperidae**							
*Agkistrodonbilineatus* (Günther, 1863)	1, 2	NT	M (11)	Pr	3	1961	UTEP:Herp:4022
*Crotalusatrox* Baird & Girard, 1853	3	LC	L (9)	Pr	2	1953	MVZ:Herp:59310
*Crotalusbasiliscus* (Cope, 1864)	1, 2, 3, 4	LC	H (16)	Pr	1	1925	CAS HERP 64974
*Crotaluslepidus* (Kennicott, 1861)	4	LC	M (12)	Pr	2	1953	MVZ:Herp:59310
*Crotalusmolossus* Baird & Girard, 1853	1, 2, 4	LC	L (8)	Pr	2	1963	KU KUH 78964
*Crotalusstejnegeri* Dunn, 1919	1	VU	H (17)	A	1	1919	Dunn 1919
**Order Testudines**							
**Chelonidae**							
*Carettacaretta* (Linnaeus, 1758)	6	VU	NE	P	5	1969	Hardy and McDiarmid 1969
*Cheloniamydas* (Linnaeus, 1758)	6	EN	NE	P	5	1960	UF Herp 39694
*Eretmochelysimbricata* (Linnaeus, 1766)	6	CR	NE	P	5	1969	Hardy and McDiarmid 1969
*Lepidochelysolivacea* (Eschscholtz, 1829)	6	VU	NE	P	5	1882	USNM Amphibians & Reptiles 211387
**Dermochelyidae**							
*Dermochelyscoriacea* (Vandelli, 1761)	6	VU	NE	P	5	1969	Hardy and McDiarmid 1969
**Emydidae**							
*Terrapenenelsoni* Stejneger, 1925	1, 2	DD	H (18)	Pr	1	1962	LACM Herps 164113
*Trachemysnebulosa* (Van Denburgh, 1895)	3	NE	H (18)	NL	1	1965	UMNH:Herp:6040
*Trachemysornata* (Gray, 1831)	1, 2	VU	H (19)	Pr	1	1831	Gray 1831
**Geoemydidae**							
*Rhinoclemmyspulcherrima* (Gray, 1855)	1, 2, 3	NE	L (8)	NL	3	1868	ANSP HRP
**Kinosternidae**							
*Kinosternonalamosae* Berry & Legler, 1980	1, 2, 3	DD	H (14)	Pr	1	1957	LACM Herps 105397
*Kinosternonhirtipes* (Wagler, 1830)	1, 2	LC	M (10)	Pr	2	1936	Taylor 1936
*Kinosternonintegrum* LeConte, 1854	1, 2, 3	LC	M (11)	Pr	1	1882	USNM Amphibians & Reptiles 12607
**Testudinidae**							
*Gopherusevgoodei* Edwards, Karl, Vaughn, Rosen, Meléndez-Torres & Murphy, 2016	1, 2, 3	VU	NE	NL	1	1963	CAS HERP 142243

**Figure 6.**
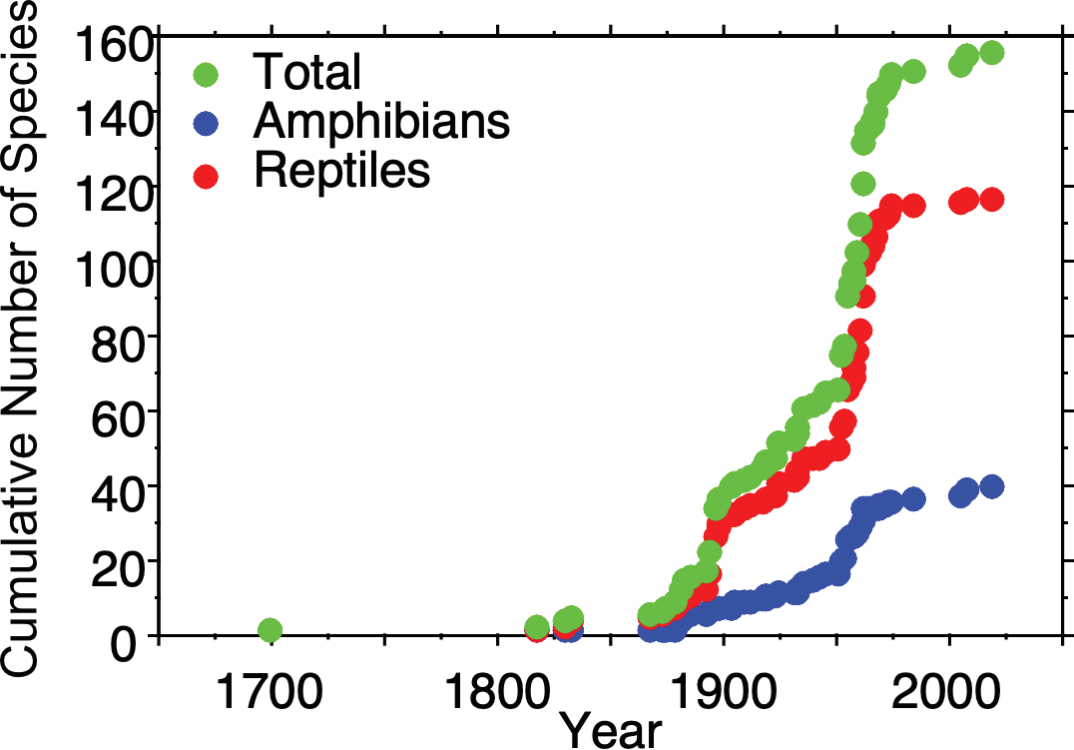
Species accumulation curves for the total herpetofauna, amphibians, and reptiles in Sinaloa, Mexico.

We suggest that there are 19 species (seven amphibians, 12 reptiles) that potentially occur in Sinaloa but that have not yet been documented in the state (Table 2). Eighteen of these species are found in Durango and Chihuahua near the border with eastern and northeastern Sinaloa, and one species is found in Nayarit near the border with southern Sinaloa. Distributional records reported in Lemos-Espinal and Smith (2007: Chihuahua), and Lemos-Espinal et al. (2019a: Durango) show that the range of these species is in close proximity to Sinaloa. Due to the relative inaccessibility of the Sierra Madre Occidental in eastern Sinaloa, and the lack of security in this region, these species have not yet been documented, but as conditions improve it is likely that they will be recorded in Sinaloa. The results of the species accumulation curves suggest that after a steep increase in the number of recorded species of amphibians and reptiles in Sinaloa during the 20^th^ century, the accumulation of newly documented species is leveling off, at least for the entire herpetofauna and for reptiles (Fig. 6). This indicates that the current checklist may be relatively complete, although the continued accumulation of amphibians suggests that there are still likely some species to be discovered in Sinaloa. Thus, we suspect that there may be some additions to the herpetofauna, including those suspected above, that will result from further survey and taxonomic work in Sinaloa in the future.

**Table 2.** List of amphibian and reptile species that potentially occur in Sinaloa.

Taxon	Explanation
**Class Amphibia**	
**Order Anura**	
**Bufonidae**	
*Anaxyruscompactilis* (Wiegmann, 1833)	Likely to occur in eastern Sinaloa
*Inciliusmccoyi* Santos-Barrera & Flores-Villela, 2011	Likely to occur in northeastern Sinaloa
**Craugastoridae**	
*Craugastortarahumaraensis* (Taylor, 1940)	Likely to occur in northeastern Sinaloa
**Eleutherodactylidae**	
*Eleutherodactyluspallidus* (Duellman, 1958)	Likely to occur in southeastern Sinaloa
**Hylidae**	
*Dryophyteswrightorum* (Taylor, 1939)	Likely to occur in northeastern Sinaloa
**Ranidae**	
*Ranachiricahuensis* Platz & Mecham, 1979	Likely to occur in eastern Sinaloa
**Order Caudata**	
**Ambystomatidae**	
*Ambystomasilvense* Webb, 2004	Likely to occur in eastern Sinaloa
**Class Reptilia**	
**Order Squamata**	
**Suborder Lacertilia**	
**Eublepharidae**	
*Coleonyxelegans* Gray, 1845	Likely to occur in southern Sinaloa
**Phrynosomatidae**	
*Sceloporusgrammicus* Wiegmann, 1828	Likely to occur in eastern Sinaloa
*Sceloporuslemosespinali* Lara-Góngora, 2004	Likely to occur in northeastern Sinaloa
*Sceloporusscalaris* Weigmann, 1828	Likely to occur in eastern Sinaloa
**Order Squamata**	
**Suborder Serpentes**	
**Dipsadidae**	
*Rhadinaealaureata* (Günther, 1868)	Likely to occur in eastern Sinaloa
**Natricidae**	
*Thamnophiseques* (Reuss, 1834)	Likely to occur in eastern-southeastern Sinaloa
*Thamnophiserrans* Smith, 1942	Likely to occur in eastern Sinaloa
*Thamnophismelanogaster* (Peters, 1864)	Likely to occur in eastern Sinaloa
*Thamnophisnigronuchalis* Thompson, 1957	Likely to occur in eastern Sinaloa
*Thamnophispulchrilatus* (Cope, 1885)	Likely to occur in eastern Sinaloa
*Thamnophisunilabialis* Tanner, 1985	Likely to occur in northeastern Sinaloa
**Viperidae**	
*Crotaluspricei* Van Denburgh, 1895	Likely to occur in eastern and northeastern Sinaloa

### General distribution

Twenty-one of the 39 species of amphibians in Sinaloa are endemic to Mexico, two of which are restricted to small areas in the Sierra Madre Occidental of Sinaloa and adjacent Durango, or Sinaloa, Nayarit, and Jalisco. Twelve are primarily distributed along the Pacific Coast and western slopes of the Sierra Madre Occidental. Two are characteristic of the Sierra Madre Occidental, and five have a widespread or spotty distribution in the Sierra Madre Occidental, central Mexico, and Sierra Madre del Sur. Of the 18 amphibian species in Sinaloa not endemic to Mexico, one is introduced, nine are found in the United States and Mexico, five are distributed from Mexico to Central or South America, and three have a wide distribution from the United States to Central or South America (Table 1). The American Crocodile (*Crocodylusacutus*) is widely distributed from southern Florida in the United States, and along the Pacific Coast of Mexico from Sonora to northern South America, including the Caribbean and the Yucatan Peninsula. Twenty of the 42 species of lizards that occur in the state are endemic to Mexico, one is endemic to Sinaloa, three are restricted to localities in the northern part of the Sierra Madre Occidental, one has a spotty distribution in Sinaloa and Colima, and one has a spotty distribution in the Pacific Coast from Sinaloa to Michoacán. Twelve species are found on the western slopes of the Sierra Madre Occidental and the Pacific Coast, one occurs in both the Sierra Madre Occidental and the Sierra Madre Oriental, and one is widely distributed in northern and central Mexico. The remaining 22 species of lizards that inhabit Sinaloa are not endemic to Mexico. Seventeen of the non-endemic species of lizards are found in the United States and Mexico, three are distributed from Mexico to Central America, and two are introduced to Sinaloa (Table 1). Thirty-four of the 64 species of snakes that inhabit Sinaloa are endemic to Mexico. Of the 30 snake species not endemic to Mexico, 19 are found in the United States and Mexico, four range from Mexico to Central or even South America, five are found from central or southern United States to Central or South America, one is a sea snake distributed across the Pacific and Indo-Pacific Oceans, and one is introduced to Sinaloa (Table 1). Six of the13 species of turtles found in Sinaloa are endemic to Mexico, one is found in the United States and Mexico, one is distributed from Mexico to Central America, and five have a circumtropical or circumglobal distribution (Table 1). One of the six endemic species of turtles that inhabit Sinaloa is shared with Baja California Sur (*Trachemysnebulosa*). This species was probably introduced in the Cape Region of Baja California by Sinaloan miners (T. Van Devender, pers. comm.).

### Habitat types

The vegetation type that hosts the highest number of amphibian and reptile species is the Tropical Deciduous Forest, which includes semi-deciduous forest, with 121 species, which represents 77.6% of the total number of species found in Sinaloa. The second highest number of amphibian and reptile species is hosted by “Thorn Forest” with 104 species, which represents 66.7% of the total species of Sinaloa. According to INEGI (2017), these two types of vegetation together occupy approximately 36% of the state. These are the two dominant vegetation types in the state, and they are also the vegetation types that originally appeared in what are now the agricultural areas of Sinaloa, which now occupy approximately 38.5% of the state surface territory (INEGI 2017). In addition, they are the types of vegetation, which due in part to their location, have been more studied from the herpetofaunistic point of view. On the other hand, the Oak Forest of Sinaloa, hosts 70 species (44.9%) of amphibians and reptiles, and the Pine-oak Forest, limited to the highest parts of the Sierra Madre Occidental of Sinaloa, hosts only 14 species (9.0%) of amphibians and reptiles of Sinaloa. Together, these two vegetation types occupy approximately 16.5% of the state surface territory (INEGI 2017). The Subtropical Mimosaceaе Cacti thorn scrub vegetation type of Sinaloa hosts 49 species (31.6%) of amphibians and reptiles. This vegetation type, limited to the northwestern tip of the state, occupies < 3.2% of the state’s territory; however, it houses a unique assortment of amphibians and reptiles, dominated by species typical of thornscrub (Bezy et al. 2017).

### Conservation status

**Figure 7.**
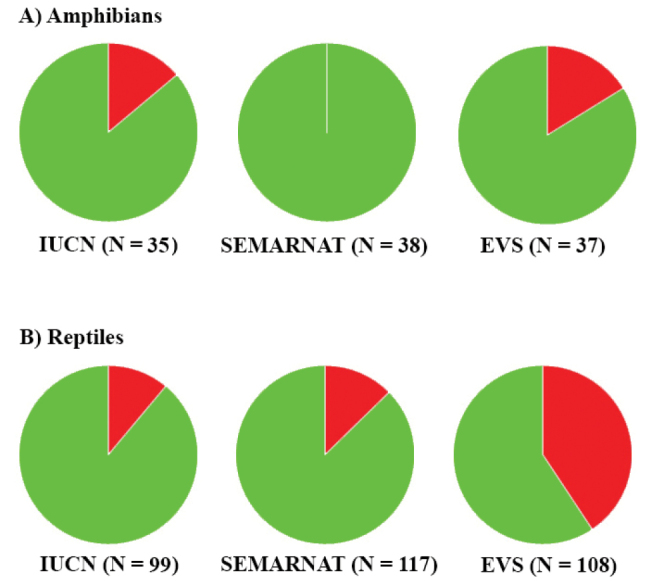
Proportion of **A** amphibians and **B** reptiles listed in protected categories on the IUCN Red List, SEMARNAT, and high EVS for Sinaloa. Green is proportion in Data Deficient and Least Concern (IUCN); Not Listed and Subject to Special Protection (we regarded the category of Subject to Special Protection in SEMARNAT equivalent to Least Concern in IUCN) (SEMARNAT); or low or medium EVS. Red is percentage in protected categories or high EVS. N is the number of species assessed.

For amphibian and reptile species together, 12.7% are IUCN listed (i.e., Vulnerable, Near Threatened, or Endangered), 9.7% are placed in a protected category (excluding NL and Pr, this last category is equivalent to the LC category of IUCN) by SEMARNAT, and 34.0% are categorized as high risk by the EVS (Table 3). For amphibians, 14.3% are IUCN listed, none are protected by SEMARNAT, and 16.2% are at high risk according to the EVS (Table 3; Fig. 7). For reptiles, 17.2% are listed by the IUCN, 12.8% are protected by SEMARNAT, and 40.2% are at high risk according to the EVS (Table 3; Fig. 7). This summary suggests that the herpetofauna of Sinaloa has relatively few species of conservation concern at a global and national scale (IUCN and SEMARNAT lists), but there might be greater conservation concerns using the EVS which is based on information specific to Mexico and Central America and so might be more likely to reflect the conservation status and needs of the Sinaloa herpetofauna. Although the SEMARNAT list is also based on information specific to Mexico, it has not been updated since 2010, and so does not take into account the numerous recent taxonomic changes and the description of new species or more recent changes in conservation status or threats. There are several taxa that, based on their IUCN listing, SEMARNAT category, or their EVS, are of conservation concern. Families with species of particular conservation concern include Craugastoridae, Eleutherodactylidae, Ambystomatidae, Crocodylidae, Dactyloidae, Eublepharidae, Helodermatidae, Iguanidae, Phrynosomatidae, Phyllodactylidae, Colubridae, Natricidae, Viperidae, Cheloniidae, and Dermochelyidae (Table 3). The status of a species in Sinaloa may differ (i.e., be worse or better) from the IUCN, SEMARNAT, and EVS assessments. Thus, assessments at the state level are needed to fully understand the conservation or management needs for the Sinaloan herpetofauna.

**Table 3.** Summary of native species present in Sinaloa by Family, Order or Suborder, and Class. Status summary indicates the number of species found in each IUCN conservation status in the order DD, LC, VU, NT, EN, CE (see Table 1 for abbreviations; in some cases species have not been assigned a status by the IUCN and therefore these may not add up to the total number of species in a taxon). Mean EVS is the mean Environmental Vulnerability Score, scores ≥ 14 are considered high vulnerability (Wilson et al. 2013a, b) and conservation status in Mexico according to SEMARNAT (2010) in the order NL, Pr, A, P (see Table 1 for abbreviations).

Scientific name	Genera	Species	IUCN	x̄ EVS	SEMARNAT
**Class Amphibia**			**DD, LC, VU, NT, EN, CE**		**NL, Pr, A, P**
**Order Anura**	**18**	**37**	**3, 26, 2, 1, 2, 0**	**9.7**	**30, 7, 0, 0**
Bufonidae	3	9	0, 7, 0, 1, 0, 0	10.6	9, 0, 0, 0
Craugastoridae	1	4	1, 2, 1, 0, 1, 0	12.3	5, 0, 0, 0
Eleutherodactylidae	1	4	2, 1, 0, 0, 1, 0	15	2, 2, 0, 0
Hylidae	7	9	0, 8, 0, 0, 0, 0	8.6	7, 2, 0, 0
Leptodactylidae	1	1	0, 1, 0, 0, 0, 0	6	1, 0, 0, 0
Microhylidae	2	3	0, 2, 0, 0, 0, 0	6.3	2, 1, 0, 0
Phyllomedusidae	1	1	0, 1, 0, 0, 0, 0	13	1, 0, 0, 0
Ranidae	1	4	0, 3, 1, 0, 0, 0	6.5	2, 2, 0, 0
Scaphiopodidae	1	1	0, 1, 0, 0, 0, 0	3	1, 0, 0, 0
**Order Caudata**	**1**	**1**	**0, 1, 0, 0, 0, 0**	**14**	**0, 1, 0, 0**
Ambystomatidae	1	1	0, 1, 0, 0, 0, 0	14	0, 1, 0, 0
**Subtotal**	**19**	**38**	**3, 27, 2, 1, 2, 0**	**9.8**	**30, 8, 0, 0**
**Class Reptilia**					
**Order Crocodylia**	**1**	**1**	**0, 0, 1, 0, 0, 0**	**14**	**0, 1, 0, 0**
Crocodylidae	1	1	0, 0, 1, 0, 0, 0	14	0, 1, 0, 0
**Order Squamata**	**56**	**103**	**7, 76, 1, 3, 0, 0**	**11.7**	**64, 29, 10, 0**
**Suborder Lacertilia**	**17**	**40**	**4, 30, 0, 1, 0, 0**	**12.9**	**27, 10, 3, 0**
Anguidae	3	3	0, 2, 0, 0, 0, 0	10.3	1, 2, 0, 0
Dactyloidae	1	2	1, 1, 0, 0, 0, 0	15	1, 1, 0, 0
Eublepharidae	1	2	0, 2, 0, 0, 0, 0	14	1, 1, 0, 0
Helodermatidae	1	2	0, 1, 0, 1, 0, 0	13	0, 0, 2, 0
Iguanidae	3	4	0, 1, 0, 0, 0, 0	14.3	3, 1, 0, 0
Phrynosomatidae	5	16	0, 14, 0, 0, 0, 0	12.8	15, 0, 1, 0
Phyllodactylidae	1	3	0, 3, 0, 0, 0, 0	12.7	2, 1, 0, 0
Scincidae	1	4	3, 1, 0, 0, 0, 0	14	2, 2, 0, 0
Teiidae	1	4	0, 4, 0, 0, 0, 0	11.8	2, 2, 0, 0
**Suborder Serpentes**	**39**	**63**	**3, 46, 1, 2, 0, 0**	**10.9**	**37, 19, 7, 0**
Boidae	1	1	0, 0, 0, 0, 0, 0	15	1, 0, 0, 0
Colubridae	22	37	2, 27, 0, 1, 0, 0	10.6	26, 7, 4, 0
Dipsadidae	8	12	1, 10, 0, 0, 0, 0	10.6	6, 6, 0, 0
Elapidae	3	3	0, 3, 0, 0, 0, 0	14.5	1, 1, 1, 0
Leptotyphlopidae	1	1	0, 0, 0, 0, 0, 0	–	1, 0, 0, 0
Natricidae	2	3	0, 2, 0, 0, 0, 0	10	2, 0, 1, 0
Viperidae	2	6	0, 4, 1, 1, 0, 0	12.2	0, 5, 1, 0
**Order Testudines**	**10**	**13**	**2, 2, 5, 0, 1, 1**	**14**	**3, 5, 0, 5**
Cheloniidae	4	4	0, 0, 2, 0, 1, 1	–	0, 0, 0, 4
Dermochelyidae	1	1	0, 0, 1, 0, 0, 0	–	0, 0, 0, 1
Emydidae	2	3	1, 0, 1, 0, 0, 0	18.3	1, 2, 0, 0
Geoemydidae	1	1	0, 0, 0, 0, 0, 0	8	1, 0, 0, 0
Kinosternidae	1	3	1, 2, 0, 0, 0, 0	11.7	0, 3, 0, 0
Testudinidae	1	1	0, 0, 1, 0, 0, 0	–	1, 0, 0, 0
**Subtotal**	**67**	**117**	**9, 78, 7, 3, 1, 1**	**11.9**	**67, 35, 10, 5**
**Total**	**86**	**155**	**12, 105, 9, 4, 3, 1**	**11.4**	**97, 43, 10, 5**

The conservation status of species found in different vegetation types in Sinaloa appear to differ (Table 1). For IUCN categories, 6.7% of the amphibian species found in the Tropical Deciduous Forest are listed in a protected category; none in the Thorn Forest of the Coastal Plains or the Subtropical Mimosaceae Cacti, 16.1% in the Oak Forest, and 33.3% in the Pine-Oak Forest. For SEMARNAT categories, no species of amphibian in Sinaloa is listed for any vegetation type (see above). For EVS, 13.3% of the amphibians in the Tropical Deciduous Forest of Sinaloa were in the high category, 5.0% in the Thorn Forest, 10.0% in the Subtropical Mimosaceae Cacti, 19.4% in the Oak Forest, and 16.7% in the Pine-oak Forest. For the IUCN listings, all five vegetation types of Sinaloa have relatively few species of reptiles in the protected categories (Tropical Deciduous Forest, 6.6%; Thorn Forest, 6.0%; Subtropical Mimosaceae Cacti, 5.1%; Oak Forest, 2.6%, and Pine-oak Forest, 0%). However, 66.7% of the reptiles in the Marine region are listed in IUCN protected categories. According to SEMARNAT, 8.8% of reptiles that inhabit the Tropical Deciduous Forest of Sinaloa, 8.3% from the Thorn Forest, 15.4% from Subtropical Mimosaceae Cacti, 10.3% from the Oak Forest, and none from the Pine-oak Forest are in protected categories. Five (83.3%) of the marine reptiles of Sinaloa are in protected SEMARNAT categories. For the Tropical Deciduous Forest of Sinaloa 38.5% of the reptile species were in the high EVS category, 38.1% in the Thorn Forest, 33.3% in the Subtropical Mimosaceae Cacti, 41.0% in the Oak Forest, and 12.5% in the Pine-oak Forest. None of the marine reptile species have been evaluated by the EVS. The marine reptiles of Sinaloa are by far the most threatened of the Sinaloa herpetofauna.

### Comparison with neighboring states

Overall, Sinaloa shares the most herpetofaunal species with Sonora, with 72.4% overlap in species (Table 4). Sinaloa shares the most amphibian species with Nayarit (78.9%). Some families, including Hylidae, Leptodactylidae, Microhylidae, Scaphiopodidae, and Ambystomatidae, show complete overlap between Sinaloa and Nayarit. The higher similarity in amphibian composition between Nayarit and Sinaloa than between Sinaloa and Sonora is due to eleven species that are shared between Sinaloa and Nayarit but not Sonora. All of these species reach their northern most distribution either in Sinaloa or Durango. The five that are shared between Sinaloa and Sonora but not with Nayarit reach their southern or southwestern most distribution in Sinaloa, and one is a species likely to occur in Nayarit. The similarity between the amphibian composition of Sinaloa and Chihuahua and Durango is lower than for Sonora and Nayarit (55.3% Durango, 52.6% Chihuahua). The amphibian species of Chihuahua and Durango have a number of species from the Chihuahuan Desert and the Sierra Madre Occidental, that do not occur in Sinaloa (Lemos-Espinal et al. 2017, 2019a). On the other hand, the Peninsula of Baja California has the lowest similarity with Sinaloa (18.1%), sharing only 28 species (Table 4), of which six are marine species with a wide global distribution. Eight of the other 22 species are only found in the northern part of the Peninsula, in the state of Baja California, far away from Sinaloa, another 10 species have a wide distribution that includes practically the entire Peninsula and the northern Mexican Pacific, four species are limited to the Baja California Peninsula and the northern part of the Mexican Pacific. Although eight to thirteen million years ago most of the Baja California Peninsula was submerged beneath the Pacific Coast and located next to the northwest coast of mainland Mexico (Grismer 2002), since its separation approximately six million years ago the fauna and flora of this peninsula has evolved under conditions of isolation, giving rise to a unique and different biota than that of Sinaloa. These numbers are an indication that Sonora, Sinaloa, and Nayarit are more similar due to the dominance of species distributed along the Pacific Coast, and Chihuahua and Durango are more similar in the composition of amphibians due to the dominance of species from the Chihuahuan Desert and Sierra Madre Occidental. The great similarity in the herpetofauna composition between Sinaloa and Sonora was also found by Enderson et al. (2009). Similarly, reptile composition is more similar between Sinaloa and its neighbors Sonora and Nayarit, than between neighbors Chihuahua and Durango. As with amphibians, Chihuahua and Durango have more reptile species from the Chihuahuan Desert and the Sierra Madre Occidental, and few species characteristic of the Pacific Coast. In addition, Sonora, Sinaloa, and Nayarit share a number of reptile species characteristic of the Pacific Coast. A greater similarity between Sonora, Sinaloa, and Nayarit is also expected by the presence of marine species in these three states, and an absence of those species in Chihuahua and Durango.

**Table 4.** Summary of the numbers of species shared between Sinaloa and neighboring Mexican states (not including introduced species). The percent of Sinaloa species shared by a neighboring state are given in parentheses. Total refers to the total number of species found in Sinaloa and four neighboring states (i.e., regional species pool) and the number in parentheses in this column is the percent of the regional species pool found in Sinaloa. – indicates either Sinaloa or the neighboring state has no species in the taxonomic group, or none of that specific taxon is shared between the states, thus no value for shared species is provided. Peninsula refers to herpetofauna of the states of Baja California and Baja California Sur pooled together.

Taxon	Sinaloa	Sonora	Nayarit	Chihuahua	Durango	Peninsula	Total
**Class Amphibia**	**38**	**25 (65.8)**	**30 (78.9)**	**20 (52.6)**	**21 (55.3)**	**3 (7.9)**	**77 (49.4)**
**Order Anura**	**37**	**24 (64.9)**	**29 (78.4)**	**19 (51.4)**	**20 (54.1)**	**3 (8.1)**	**67 (55.2)**
Bufonidae	9	8 (88.9)	5 (55.6)	6 (66.7)	7 (77.8)	2 (22.2)	17 (52.9)
Craugastoridae	5	2 (40)	4 (80)	1 (20)	3 (60)	–	6 (83.3)
Eleutherodactylidae	4	1 (25)	2 (50)	1 (25)	2 (50)	–	6 (66.7)
Hylidae	9	4 (44.4)	9 (100)	3 (33.3)	4 (44.4)	–	14 (64.3)
Leptodactylidae	1	1 (100)	1 (100)	–	–	–	1 (100)
Microhylidae	3	2 (66.7)	3 (100)	2 (66.7)	–	–	4 (75)
Phyllomedusidae	1	1 (100)	1 (100)	1 (100)	1 (100)	–	1 (100)
Ranidae	4	4 (100)	3 (75)	4 (100)	2 (50)	–	14 (28.6)
Scaphiopodidae	1	1 (100)	1 (100)	1 (100)	1 (100)	1 (100)	4 (25)
**Order Caudata**	**1**	**1 (100)**	**1 (100)**	**1 (100)**	**1 (100)**	–	**10 (10)**
Ambystomatidae	1	1 (100)	1 (100)	1 (100)	1 (100)	–	4 (25)
Plethodontidae	–	–	–	–	–	–	6 (0)
**Class Reptilia**	**117**	**87 (74.4)**	**76 (65)**	**64 (54.7)**	**57 (48.7)**	**25 (21.4)**	**358 (32.7)**
**Order Crocodylia**	**1**	**1 (100)**	**1 (100)**	–	–	–	**1 (100)**
Crocodylidae	1	1 (100)	1 (100)	–	–	–	1 (100)
**Order Squamata**	**103**	**75 (72.8)**	**66 (64.1)**	**58 (57.3)**	**55 (53.4)**	**19 (18.4)**	**332 (31)**
**Suborder Amphisbaenia**	–	–	–	–	–	–	**1 (0)**
Bipedidae	–	–	–	–	–	–	1 (0)
**Suborder Lacertilia**	**40**	**27 (67.5)**	**20 (50)**	**18 (45)**	**21 (52.5)**	**7 (17.5)**	**177 (22.6)**
Anguidae	3	1 (33.3)	2 (66.7)	2 (66.7)	3 (100)	–	11 (27.3)
Anniellidae						–	2 (0)
Corytophanidae	–	–	–	–	–	–	1 (0)
Crotaphytidae	–	–	–	–	–	–	8 (0)
Dactyloidae	2	1 (50)	1 (50)	1 (50)	1 (50)	–	2 (100)
Eublepharidae	2	2 (100)	–	–	1 (50)	1 (50)	6 (33.3)
Helodermatidae	2	2 (100)	1 (50)	1 (50)	1 (50)	–	2 (100)
Iguanidae	4	2 (50)	2 (50)	1 (25)	1 (25)	2 (50)	12 (33.3)
Phrynosomatidae	16	12 (75)	8 (50)	9 (56.3)	11 (68.8)	3 (18.8)	72 (22.2)
Phyllodactylidae	3	2 (66.7)	2 (66.7)	1 (33.3)	1 (33.3)	–	9 (33.3)
Scincidae	4	2 (50)	2 (50)	2 (50)	1 (25)	–	16 (25)
Teiidae	4	3 (75)	2 (50)	1 (25)	1 (25)	1 (25)	30 (13.3)
Xantusidae	–	–	–	–	–	–	6 (0)
**Suborder Serpentes**	**63**	**48 (76.2)**	**46 (73.0)**	**41 (65.1)**	**34 (54.0)**	**12 (19.0)**	**154 (40.9)**
Boidae	1	1 (100)	1 (100)	1 (100)	1 (100)	–	3 (33.3)
Colubridae	37	28 (75.7)	23 (62.2)	23 (62.2)	21 (56.8)	7 (18.9)	74 (50)
Dipsadidae	12	7 (58.3)	11 (91.7)	6 (50)	4 (33.3)	2 (16.7)	24 (50)
Elapidae	3	3 (100)	3 (100)	2 (66.7)	–	1 (33.3)	5 (60)
Leptotyphlopidae	1	1 (100)	1 (100)	1 (100)	–	–	4 (25)
Loxocemidae	–	–	–	–	–	–	1 (0)
Natricidae	3	3 (100)	3 (100)	3 (100)	3 (100)	1 (33.3)	16 (18.8)
Viperidae	6	5 (83.3)	4 (66.7)	5 (83.3)	5 (83.3)	1 (16.7)	27 (22.2)
**Order Testudines**	**13**	**11 (84.6)**	**9 (69.2)**	**5 (38.5)**	**2 (15.4)**	**6 (46.2)**	**25 (52)**
Cheloniidae	4	4 (100)	3 (75)	–	–	4 (100)	4 (100)
Dermochelyidae	1	1 (100)	1 (100)	–	–	1(100)	1(100)
Emydidae	3	2 (66.7)	2 (66.7)	1 (33.3)	–	1 (33.3)	8 (37.5)
Geoemydidae	1	1 (100)	1 (100)	1 (100)	–	–	1 (100)
Kinosternidae	3	2 (66.7)	2 (66.7)	2 (66.7)	2 (66.7)	–	8 (37.5)
Testudinidae	1	1 (100)	–	1 (100)	–	–	3 (66.7)
**Total**	**155**	**112 (72.3)**	**106 (68.4)**	**84 (54.2)**	**78 (50.3)**	**28 (18.1)**	**435 (35.6)**

## Appendix 1

Museum collections included in the VertNet.org database records of Sinaloa amphibians and reptiles that house specimens of the first record of a species in Sinaloa.

**AMNH** Collection of Herpetology, Herpetology Department, American Museum of Natural History

**ANSP** Academy of Natural Sciences of Philadelphia. ANSP Herpetology

**CAS** Collection of Herpetology, Herpetology Department, California Academy of Sciences

**CMNH** Collection of Herpetology, Amphibian and Reptile Section, Carnegie Museum of Natural History, Pittsburgh

**FMNH** Division of Amphibians and Reptiles, Field Museum of Natural History

**FSM-UF** Collection of Herpetology, Florida State Museum, University of Florida

**LACM** LACM">Collection of Herpetology, Herpetology Section, Natural History Museum of Los Angeles County

**MCZ** Collection of Herpetology, Museum of Comparative Zoology, Harvard University Cambridge

**MNHUK** Museum of Natural History, Division of Herpetology, University of Kansas

**MSUM** Michigan State University Museum. MSUM Ichthyology and Herpetology Collections

**SDNHM** Collection of Herpetology, Herpetology Department, San Diego Natural History Museum

**TCWC** Collection of Herpetology, Texas Cooperative Wildlife Collection, Texas A&M University

**TNHC** Collection of Herpetology, Texas Natural History Collection, University of Texas Austin

**UAZ** Amphibians and Reptiles Collections, University of Arizona

**UCM** Collection of Herpetology, University of Colorado Museum

**UMNH** Natural History Museum of Utah. UMNH Reptiles and Amphibians Collection

**UMMZ** Collection of Herpetology, Museum of Zoology, University of Michigan Ann Arbor

**USNM** Collection of Herpetology, Department of Vertebrate Zoology, National Museum of Natural History, Smithsonian Institution

**UTAMM** Merriam Museum, University of Texas Arlington

**UTEP** Collection of Herpetology, Laboratory of Environmental Biology, Biological Science Department, University of Texas – El Paso
